# Molecular Dynamics Simulations of HEMA-Based Hydrogels for Ophthalmological Applications

**DOI:** 10.3390/molecules29235784

**Published:** 2024-12-07

**Authors:** Katarzyna Filipecka-Szymczyk, Malgorzata Makowska-Janusik, Wojciech Marczak

**Affiliations:** Faculty of Science and Technology, Jan Dlugosz University, Al. Armii Krajowej 13/15, 42-200 Czestochowa, Poland; k.filipecka-szymczyk@ujd.edu.pl (K.F.-S.); m.makowska@ujd.edu.pl (M.M.-J.)

**Keywords:** polymers, glass transition, diffusion, free volume, hopping mechanism, subdiffusion, contact lens

## Abstract

The structural and dynamic properties of poly(2-hydroxyethyl methacrylate) (PHEMA) and poly(*N*-vinylpyrrolidone-*co*-2-hydroxyethyl methacrylate) [P(VP-*co*-HEMA)], dry and as hydrogels, were studied by molecular dynamics simulations. The P(VP-*co*-HEMA) chains differed in the number of VP mers, distributed randomly or in blocks. In all considered configurations, HEMA and VP side chains proved relatively rigid and stable. Water concentration had a significant impact on their dynamic behavior. Oxygen atoms of hydroxyl and carbonyl groups of HEMA and carbonyl groups of VP are preferred sites of hydrogen bonding with water molecules. The copolymer swelling results in diffusion channels, larger in systems with high water content. In low-hydrated materials, water shows subdiffusion, while normal diffusion predominates in the high-hydrated ones. The VP side chains in copolymers with HEMA do not enhance the mobility of water.

## 1. Introduction

Hydrogels, due to their unique properties, are applied as biomaterials in medicine, pharmacy, and tissue engineering [1,2,3,4,5,6]. Hydrogel-based contact lenses show several advantages over traditional glasses. By adhering directly to the cornea, they do not cause image distortion, do not limit the field of vision, and, last but not least, are very lightweight. However, contact lenses are not without drawbacks. Despite the intensive improvement of materials used in their production, many patients still decide to shorten their wearing time or even completely abandon this type of correction, primarily due to persistent discomfort caused by bacterial or fungal infections, pathological changes, or other complications [7,8,9]. Additionally, long-term hypoxia and hypercapnia, which can occur when wearing lenses with insufficient oxygen permeability, may lead to serious health consequences [10].

One of the synthetic hydrogels widely used for contact lenses is poly(2-hydroxyethyl methacrylate) (PHEMA). PHEMA is cell- and blood-compatible in particular, and virtually non-cytotoxic and non-thrombogenic [11,12]. Hydroxyl and carbonyl groups of HEMA are capable of forming hydrogen bonds with water molecules. Hydrophobic methyl groups contribute to the hydrolytic stability and mechanical strength of the polymer matrix. Moreover, PHEMA is inert to many chemicals, resistant to degradation, thermally stable, and has desirable mechanical properties [13,14,15]. However, contact lenses based on hydrated PHEMA polymers may contain only up to 38% water by mass [16,17]. The excessively low water content impairs the oxygen supply to the cornea. Therefore, PHEMA is copolymerized with various hydrophilic species in hopes of obtaining a better water-absorbing material with higher oxygen permeability [16,18,19]. Due to nitrogen and oxygen atoms of the lactam ring, *N*-vinylpyrrolidone (VP) is a common hydrophilic component of the copolymer matrix and an internal wetting agent [20,21]. Copolymers based on the VP and HEMA form hydrogel contact lens material containing 40–60% water by mass [22].

Water diffuses through hydrogel contact lenses in two directions: (i) from the surface of the cornea to the front surface of the contact lens, where it evaporates, and (ii) from the front surface of the lens into the space beneath due to osmotic pressure or pressure from the eyelids. The first leads to undesirable dehydration of the lens that may cause drying of the cornea and the lens sticking to the eyeball surface. The second provides an adequate tear film underneath the lens, which is essential for lens movement and removal of metabolic debris [23,24,25]. In hydrogel lenses, water is also the main oxygen carrier but this phenomenon is not fully explained.

The aim of the present work is to attempt to explain water’s role in oxygen transport by its mobility studies performed using the Molecular Dynamics (MD) simulation method. MD simulations of polymers reveal relationships between the microscopic structure of the material and its macroscopic properties. Computer modeling can help in optimizing the costly chemical syntheses and facilitate the interpretation of the experimental results. Despite this, MD simulations of hydrogels for potential ophthalmological applications are rather scarce.

This work deals with PHEMA and P(VP-*co*-HEMA) hydrogels, the interactions of water molecules with the polymer matrix in particular. We focused the studies on water transport in the hydrogels, which is determined by the copolymer side chains’ properties and their arrangement. The water permeability of hydrogels is one of the material characteristics crucial in ophthalmological applications.

## 2. Results and Discussion

**Figure 1:**
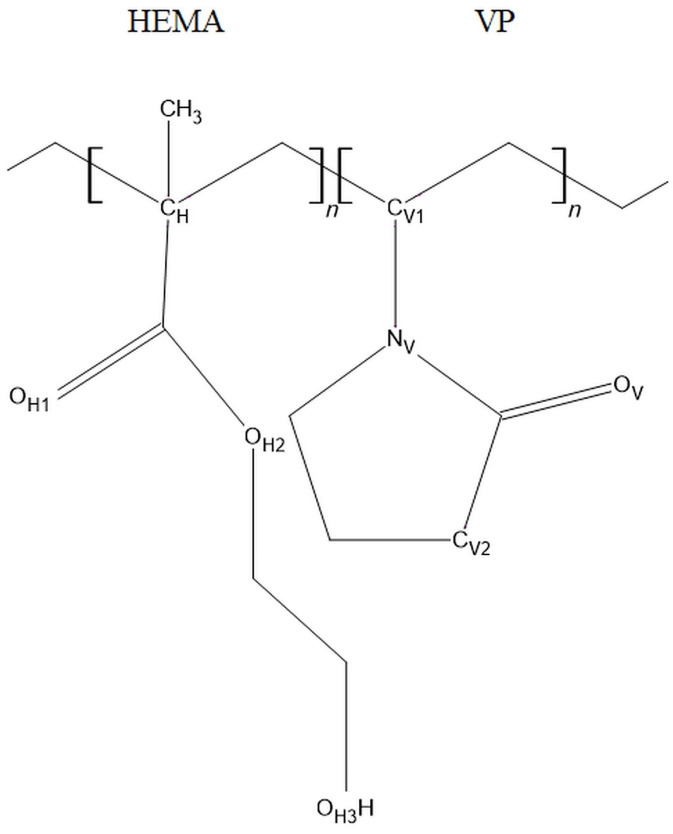
A fragment of the P(VP-*co*-HEMA) polymer chain.

Using the molecular dynamics approach (MD), we simulated various molecular interactions involving polymer chains and water. To keep this report readable, we applied the following abbreviations. HEMA stands for 2-hydroxyethyl methacrylate, VP for *N*-vinylpyrrolidone, “-*co*-“ between HEMA and VP means “copolymer” and the prefix “P” means “poly”. For example, P(VP-*co*-HEMA) denotes poly(*N*-vinylpyrrolidone-*co*-2-hydroxyethyl methacrylate), while PHEMA denotes poly(2-hydroxyethyl methacrylate). Figure 1 shows a fragment of the P(VP-*co*-HEMA) chain with atoms labeled for further reference in the paper. From here on, the subscripts H, V, and W placed on the right of chemical symbols identify atoms belonging to HEMA, VP, and water molecules, respectively.

We studied dry and hydrated PHEMA and P(VP-*co*-HEMA). The hydrogels contained 10, 20, 40, and 60% of water by mass. All polymer chains were composed of 50 mers. The P(VP-*co*-HEMA) copolymers consisted of 13 mers of one kind and 37 of the other, in block or random configurations. The following five polymers were studied:

- homopolymer PHEMA (H50);
- block copolymer V_13_H_37_ (B37);
- block copolymer V_37_H_13_ (B13);
- random copolymer with 13 VP and 37 HEMA mers (R37);
  H_2_V_5_H_2_V_6_H_2_V_2_HV_10_HVH_2_V_6_HV_6_H_2_V;
- random copolymer with 37 VP and 13 HEMA mers (R13);
  HV_2_H_8_V_3_H_3_V_2_H_2_VH_6_VH_10_V_2_H_4_VH_3_V.

V stands for VP and H for HEMA, and the subscript denotes the number of mers. Codes in parentheses identify the polymers and will be used for simplicity from now on.

In this paper, we compare the simulation results with those reported earlier for dry and hydrated poly(2-methacryloyloxyethyl phosphorylcholine-*co*-2-hydroxyethyl methacrylate) [26]. Rather than the full name of that polymer, we shall use the abbreviation P(MPC-*co*-HEMA) in the following parts of this paper.

### 2.1. Glass Transition Temperatures

**Figure 2:**
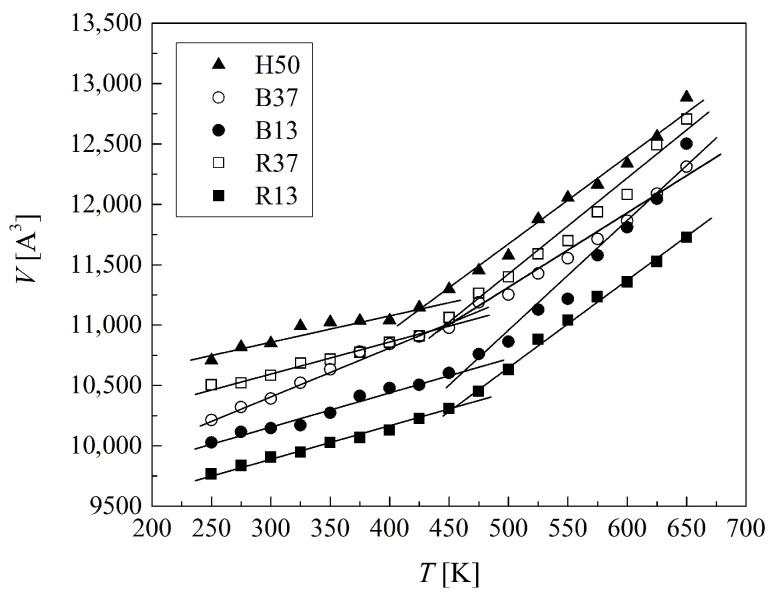
Volume versus temperature relationships for the non-hydrated polymers, fitted by bilinear functions.

The glass transition temperatures *T*_g_ of the non-hydrated polymers were assessed from the temperature dependencies of the volume plotted in Figure 2. At *T*_g_, a step change in the thermal expansion of the system occurs, ${\left(\partial V/\partial T\right)}_{P}$, which is manifested in a break of the volume–temperature line. These distinct breaks evidenced that the simulated polymer sizes were large enough to show the glass transition [27]. The values of *T*_g_ are collected in Table 1.

The VP mers affect the glass transition temperature. The more VP mers in the copolymer chain, the higher the *T*_g_ value. Block copolymers showed higher *T*_g_ than the random ones and the effect of the mer sequence was even more pronounced than that of the VP mers fraction. The differences in *T*_g_ were 7 to 8 K between the copolymers with 13 and 37 VP mers, while they were 10 to 11 K between the block and random configuration.

The experimental glass transition of PHEMA lies within the temperature range from 323 K to 401 K [28,29,30,31,32,33,34,35], while *T*_g_ = 425 K for the simulated H50 (Table 1). Although the latter temperature is higher indeed, the difference is rather small in comparison with the width of the experimental temperature interval. The discrepancy may be due to impurities in the experimental samples. For example, traces of water in the polymer material result in the lowered glass transition temperature. Such traces were detected even after several months of drying [36]. On the other hand, the discrepancy can be explained by the differences in size between the simulated and real polymer chains, as well as the intrinsic features of the simulation procedure. Note that the glass transition temperatures assessed from simulations are generally higher than experimental ones [27,37]. Differences in the polymers’ morphology doubtless affect the *T*_g_. The simulated polymers are fully amorphous, while the real samples may be locally ordered with macroparticles even cross-linked, which limits the motions of polymer chains.

PVP showed the experimental glass transition temperature in the range from 327 K to 469 K [34,38,39,40,41,42], higher than that of 323 K to 401 K of PHEMA [28,29,30,31,32,33,34,35]. This was explained as a consequence of the rigid pyrrolidone group in side chains [38,39,40,41,42]. Similarly, as for homopolymers, the experimental *T*_g_ of the P(VP-*co*-HEMA) may be recorded in a wide temperature range [34,43]. The glass transition temperature of a copolymer is usually between those of the corresponding homopolymers. Some block copolymers show two values of *T*_g_ characteristic of the two homopolymers [44,45]. Our simulations did not reveal such a phenomenon.

The *T*_g_ was simulated to check the correctness of the force field selection. The obtained tendency of the *T*_g_ changes is in agreement with the above-discussed experimental data. Additionally, performing the MD simulations at the temperature of the human body, we are sure that the atomic system is in the glassy state as it is in real experiments.

### 2.2. Structure and Chain Packing

A rigidity of HEMA and VP mers is manifested in radial distribution functions (RDFs) for pairs of atoms belonging to the same side chain. For both mers, the first atom for which the RDF was calculated is that of carbon in the main polymer chain, C_H_ and C_V1_ in Figure 1. The second atom is at the end of the respective side chain: the O_H3_ of HEMA and C_V2_ of VP.

**Figure 3:**
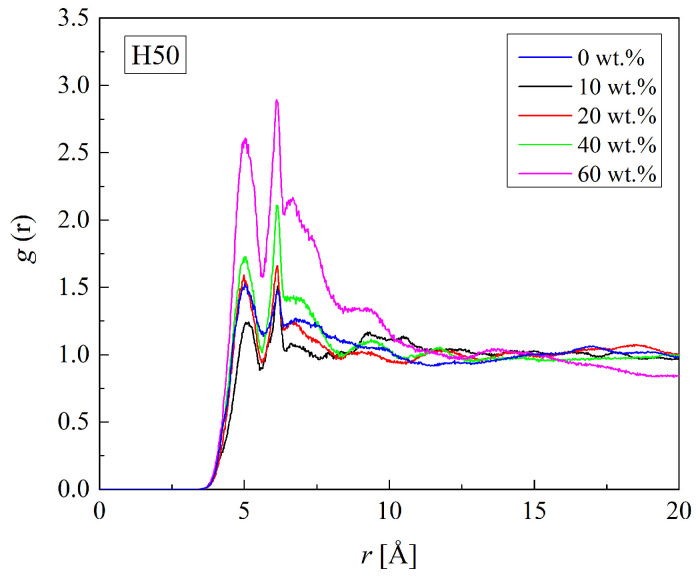
Radial distribution function *g*(*r*) of the C_H_-O_H3_ pair of HEMA in the H50 polymer with various water content.

**Figure 4:**
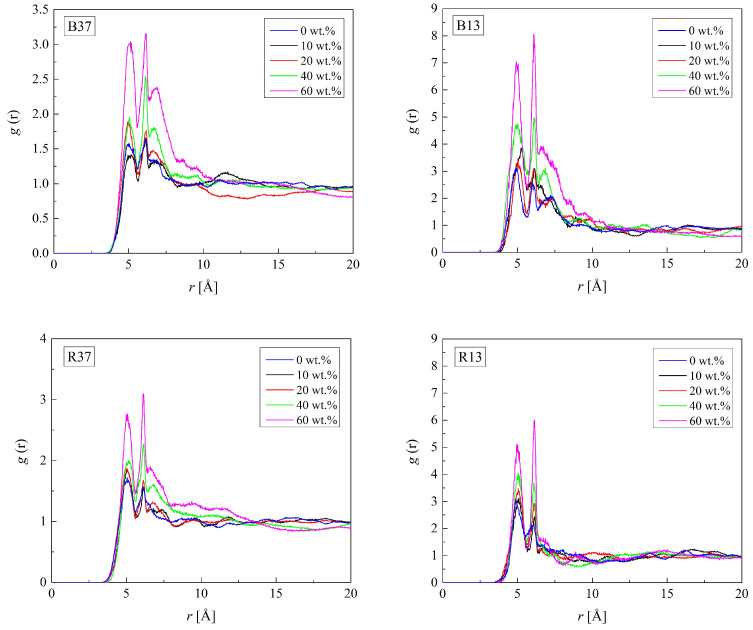
Radial distribution function *g*(*r*) of the C_H_-O_H3_ pair of HEMA in the B37, B13, R37, and R13 copolymers with various water content.

The RDFs of the C_H_-O_H3_ pairs in H50, dry and hydrated, are shown in Figure 3. The shortest distance between C_H_ and O_H3_ atoms indicated by the first RDF peak is equal to 5.0 Å. It remains the same for the hydrated PHEMA. Figure 4 illustrates that neither the arrangement of mers in the copolymer chain nor the hydration affects the C_H_-O_H3_ distance significantly. Higher RDF peaks for hydrated polymers are due to the lower number density of HEMA mers in the cell expanded to accommodate water molecules (cf. Table 2). Thus, HEMA mers are rigid, like those in P(MPC-*co*-HEMA) [26].

Also, the RDFs for the C_V1_-C_V2_ pair in VP of P(VP-*co*-HEMA) were calculated. All the RDFs are of similar shape with a well-defined peak at 3.8 Å. Neither the arrangement of the copolymer chain nor the hydration affects the VP geometry because of the rather stiff pyrrolidine ring in the mer. The RDFs evidence that PHEMA and P(VP-*co*-HEMA) polymers are stable systems with rigid side groups, and significantly stiffer than that of MPC studied previously [26].

### 2.3. Distribution of Water Molecules

**Figure 5:**
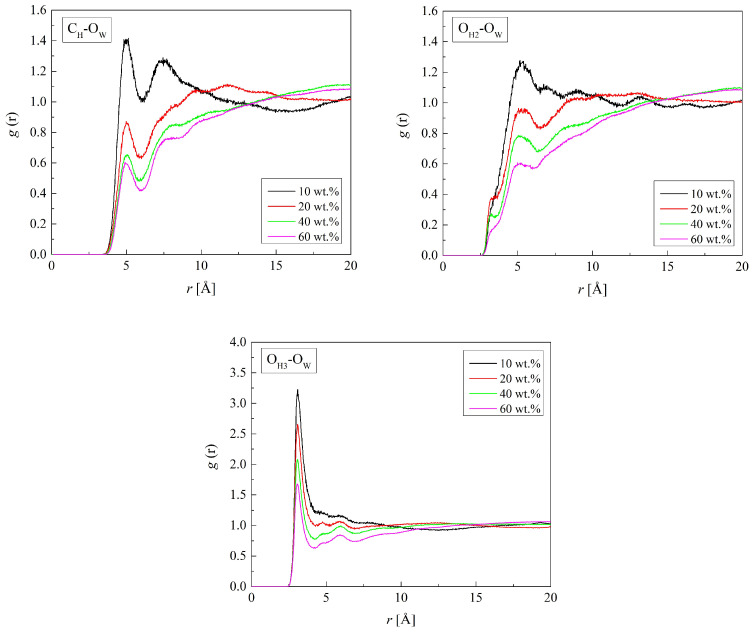
Radial distribution function *g*(*r*) for atom pairs: water oxygen (O_W_) and C_H_, O_H2_, and O_H3_ of HEMA for the H50 polymer with various water content.

**Figure 6:**
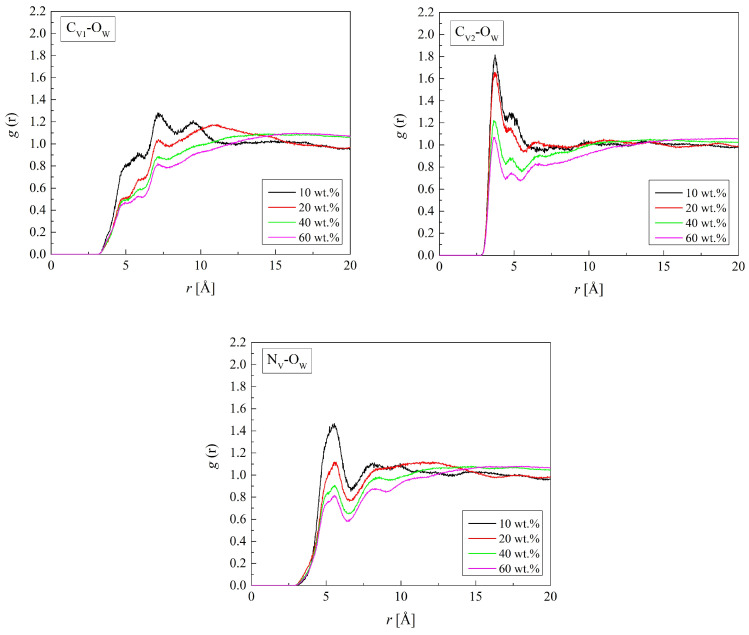
Radial distribution function *g*(*r*) for atom pairs: water oxygen (O_W_) and C_V1_, C_V2_, and N_V_ of VP for the B13 copolymer with various water content.

Radial distribution functions show where water molecules gather in the hydrogels. The first peaks of the RDFs for the atoms of polymer chains and oxygen atoms of water (O_W_) are well defined (Figure 5 and Figure 6). The RDF shapes with one distinct peak suggest an amorphous polymer environment with a rather short-range order of water molecules. Some RDFs, however, show second peaks evidencing the second hydration shell. At distances beyond the second hydration sphere, water molecules are distributed randomly.

The RDFs of the C_H_-O_W_, O_H2_-O_W,_ and O_H3_-O_W_ pairs calculated for H50 are plotted in Figure 5. The RDFs for B37, B13, R37, and R13 are similar to the presented ones. The shortest distance between O_H3_ and O_W_ is 3.1 Å and remains constant regardless of the water content in the system within the studied hydration range. Similarly, the shortest interatomic distance in the O_H2_-O_W_ pair is 3.3 Å, although the corresponding *g*(*r*) peak is small and a prominent peak appears only at 5.2 Å. The two peaks at ca. 3 Å distances suggest hydrogen bonds between water molecules and the ether and hydroxyl groups of the HEMA chain. Hydrogen bonds involving the terminal hydroxyl group are favored over those with the ether oxygen because of the limited access to the latter caused by the main as well as neighboring side chains of the polymer. The O_H2_-O_W_ and C_H_-O_W_ distances of ca. 5 Å are typical of non-hydrogen-bonded molecules. Similar results were obtained for P(MPC-*co*-HEMA) copolymers [26].

Figure 6 shows the RDF for C_V1_-O_W_, C_V2_-O_W_, and N_V_-O_W_ pairs calculated for the hydrated B13. The RDFs obtained for the other P(VP-*co*-HEMA) copolymers are similar to the reported ones. The shortest distance, 3.7 Å, is between C_V2_ and O_W_. At this distance, the RDFs for the C_V1_-O_W_ pair do not show a peak, but just a bump at ca. 5 Å and a peak only at ca. 7 Å. That suggests much weaker interactions of the polymer main chain with water molecules. The distance between N_V_ and O_W_ is equal to 5.6 Å.

### 2.4. Hydrogen Bonding

**Figure 7:**
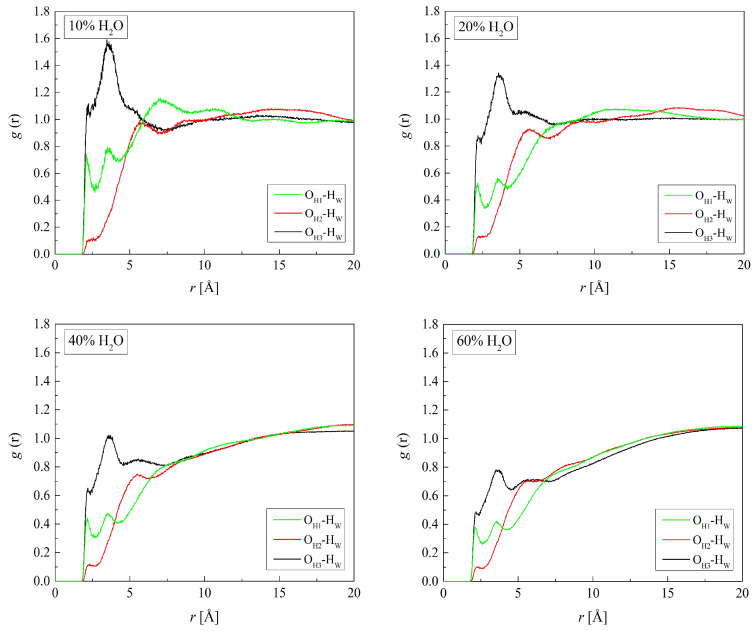
Radial distribution function *g*(*r*) for atom pairs: water hydrogen and HEMA oxygen for H50 polymer with various water content.

Interactions between polymer and water molecules, hydrogen bonds, and electrostatic interactions, in particular, determine the properties of the gel [46,47]. In the simulations, we considered all potential hydrogen-bonding sites: the oxygen and nitrogen atoms in HEMA and VP mers, as well as the hydrogen and oxygen atoms of water molecules. Figure 7 shows the RDF for the three pairs: (O_H1_, O_H2_, or O_H3_ in H50)-H_W_. The distance O_H1_-H_W_, manifested in the first peaks of the RDF, is 2.2 Å. That indicates hydrogen bonds in which the O_H1_ atoms are acceptors of the hydrogen atoms of water molecules. Just a small peak occurs on the O_H3_-H_W_ RDF at 2.2 Å. This seems to contradict the idea of hydrogen bonds between O_H3_ and O_W_ suggested in the previous section. However, the hydroxyl group of HEMA can be a hydrogen donor rather than an acceptor, which would be indeed unconventional behavior, as long-chain alcohols are worse proton donors than water. Usually, water is regarded as more acidic than alcohols; however, this does not apply to the gaseous phase, for which a partially reversed order was reported [48,49]. Results of more recent studies also do not rule out alcohol–water hydrogen bonds where alcohol molecules are donors [50]. Whether it is the interaction nature or just a simulation artefact cannot be resolved at this stage of the study. The lack of a distinct peak may also be explained by the mobility of HEMA side groups as well as the hydrogen atoms. This seems, however, less probable because of the pronounced second peak at the distance of 3.5 Å. The latter evidences the second hydration layer surrounding the hydroxyl and carbonyl HEMA oxygen atoms due to water–water hydrogen bonds.

**Figure 8:**
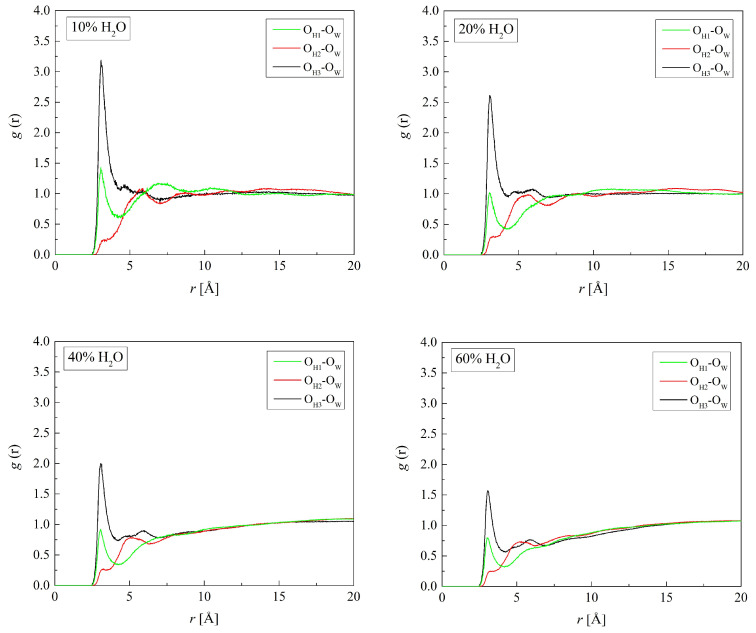
Radial distribution function *g*(*r*) for atom pairs: water oxygen and HEMA oxygen for H50 polymer with various water content.

The sharp peaks at 3.1 Å of the O_H3_-O_W_ RDF similar to those of the O_H1_-O_W_ ones plotted in Figure 8 confirm the hydrogen bonds between O_H3_ and O_H1_ of HEMA atoms and water molecules. The radial distribution functions for O_H2_-H_W_ as well as O_H2_-O_W_ do not show distinct peaks. That suggests weaker, if any, hydrogen bonds between the ether O_H2_ and water molecules. Thus, the hydroxyl and carbonyl groups are preferred sites for hydrogen bonding. These functional groups make PHEMA compatible with water. Experimental studies of water transport in crosslinked HEMA led to a similar conclusion [51]. Water in excess disrupts the hydration spheres of the HEMA hydroxyl and carbonyl groups. This is manifested in vanishing oscillations of the RDFs at distances beyond those of the second peak. Such behavior also showed other hydrated polymers [52,53], aqueous P(MPC-*co*-HEMA) in particular [26].

**Figure 9:**
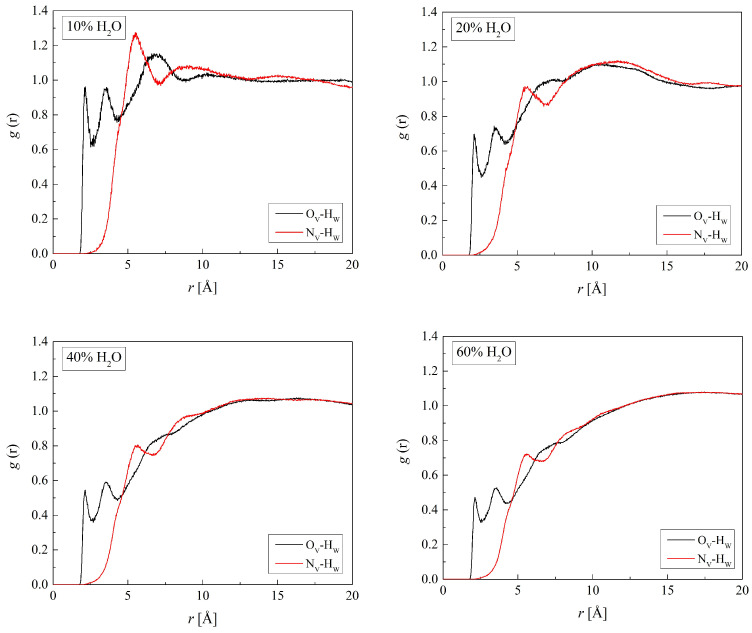
Radial distribution function *g*(*r*) for atom pairs: water hydrogen and VP oxygen or nitrogen for B13 copolymer with various water content.

**Figure 10:**
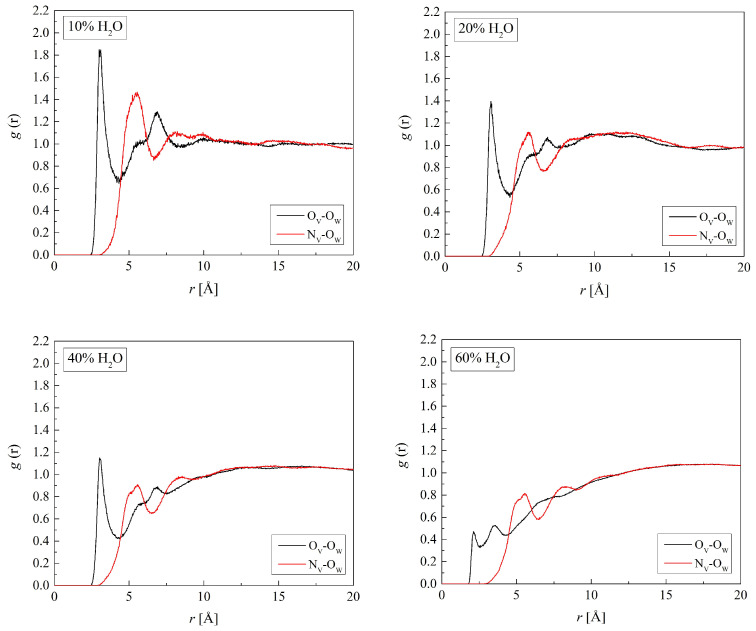
Radial distribution function *g*(*r*) for atom pairs: water oxygen and VP oxygen or nitrogen for B13 copolymer with various water content.

Figure 9 and Figure 10 show the RDFs for the pairs (O_V_ or N_V_ of B13)-(H_W_ or O_W_). The respective RDFs for the three other copolymers are similar. The first peaks of the RDFs at 2.2 Å for O_V_-H_W_ and 3.1 Å for O_V_-O_W_ suggest water molecules hydrogen-bonded to the carbonyl group of VP forming the first hydration shell. The second peaks of the RDFs evidence the second hydration shell. A significant distance of 5.6 Å between the N_V_ atom and the nearest water molecules indicates that hydrogen bonds do not occur there despite the strong hydrogen acceptor properties of the nitrogen atom. The neighboring side chains form steric hindrances that keep water molecules away from the electron lone pair of the nitrogen atom.

Along with the increasing water concentration, the first peaks of the O_V_-H_W_ and O_V_-O_W_ RDFs of all studied hydrogels diminish. That evidences the equilibrium between the O_V_ hydration and bulk water shifted towards the latter. Initially, water saturates potential hydrogen bonding sites in the polymers, forming the “hydration water” in their vicinity. Gradually, the number of excess water molecules increases and clusters of non-hydration water arise which resemble the bulk phase. Other hydrated polymers showed similar behavior [26,52,54].

### 2.5. Free Volumes

**Figure 11:**
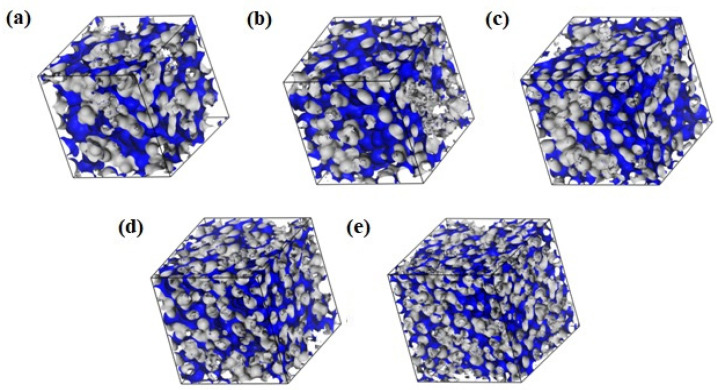
Morphology of free volumes in H50 polymer, dry (**a**) and with different water content: 10% (**b**), 20% (**c**), 40% (**d**), and 60% (**e**) by mass for the probe of infinitesimal radius. The walls of the empty channels making up the free volumes are marked in blue.

The size and distribution of free volumes determine the intrapolymer transport of fluids. The fractional free volumes (FFVs) for twenty-five systems calculated with a probe of null radius (a point probe) are reported in Table 3. Figure 11 shows example morphologies for the dry and hydrated H50. Morphologies of the copolymers are similar to these and do not depend significantly on the polymer built. The FFVs in B13 and R13 are slightly smaller than those in H50, B37 and R37. On hydration, the FFVs decrease (Table 3) and the free volumes disperse (Figure 11).

**Figure 12:**
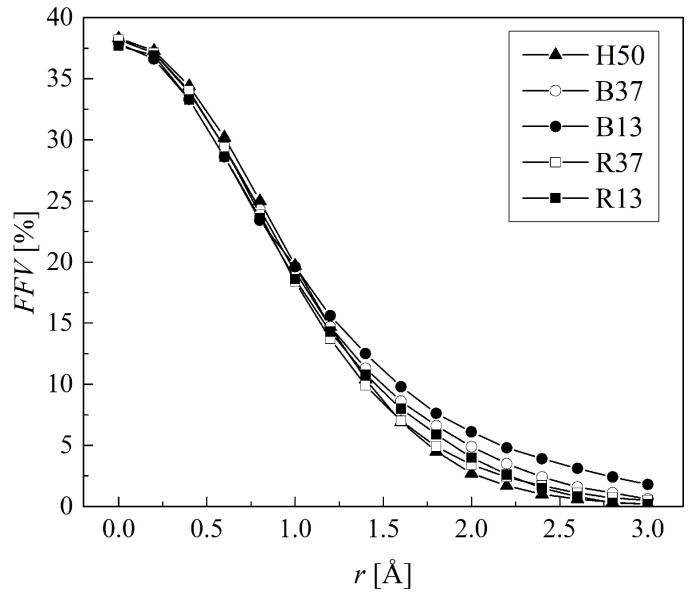
The fractional free volumes (FFVs) in PHEMA and P(VP-*co*-HEMA) determined with probes of various radii.

Figure 12 shows the FFV of non-hydrated hydrogels for test probes of different radii. The free volumes too small for water molecules cannot influence the transport of ophthalmological important substances within the studied material [55]. Indeed, only sufficiently big free volumes are important from this point of view. Therefore, a water molecule, represented by a sphere with a radius of 1.4 Å [56], was chosen as the probe. In these simulations, the polymers’ geometries remained as they were when swelled, while the water molecules were removed from the unit cells. The results are collected in Table 4. Free volumes with a diameter smaller than the probe molecule were neglected. In dry B37 and B13 copolymers, the FFVs available for water are bigger for block than random configuration and bigger than for H50. However, the differences are relatively small and vanish for higher water concentrations.

**Figure 13:**
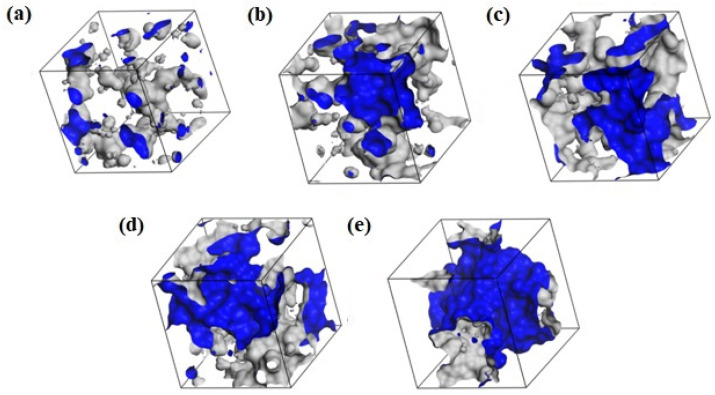
The volume in the simulated H50 polymer available for or filled with water: dry (**a**) and with different water content: 10% (**b**), 20% (**c**), 40% (**d**), and 60% (**e**) by mass. The walls of polymer channels available for the probe with a radius of 1.4 Å (i.e., a “water molecule”) are marked in blue.

Examples of the free volume morphology in H50 are illustrated in Figure 13. A part of the free volume results from the non-optimal packing and thermal motions of polymer chains. Hydration increases the free volume around the polymer chains. Concurrently, separated free volumes merge into diffusion channels. In 40 and 60% hydrogels, water is not only absorbed in the polymer matrix but also gathers around the chains. This “free” water does not interact with the polymer chains directly but rather resembles the bulk water. This simulation result is consistent with experiments suggesting bulk water in hydrates containing at least 30% of water by mass [57,58,59].

The present simulation results confirm that mobile and flexible chains facilitate the formation of diffusion channels crucial for intramaterial transport reported in [34,36]. Penetrant water molecules diffuse into the voids between the side chains in subnanometric, random “jumps”. Thermally induced movements of the polymer chains favor the creation of spacious voids which make jumps from one to another longer and more frequent, as suggested in [44,45]. VP mers in the copolymer chain decrease the free volume of the system independently of their sequence.

### 2.6. Mobility and Dynamics of the Polymer Chains

**Figure 14:**
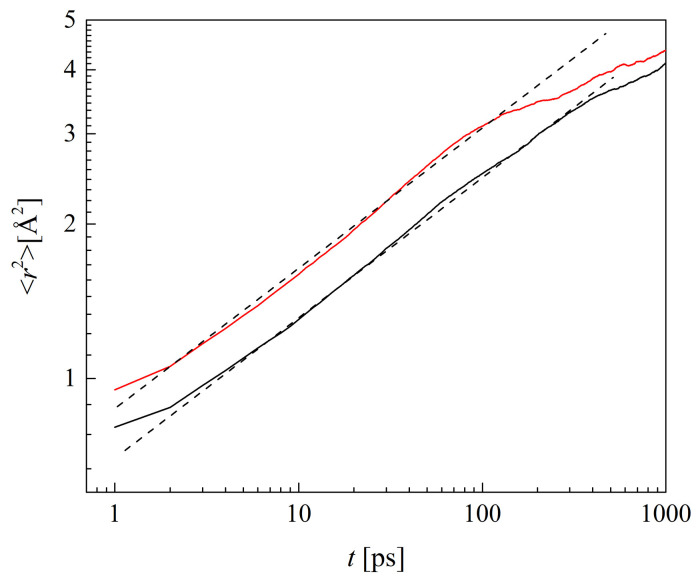
Mean square displacements of C_V2_ atoms in dry copolymers as a function of time. Solid lines: simulation results for R13 (red) and B13 (black); dotted lines: fitted Equation (1).

The mean square displacement (MSD) characterizes the mobility of polymer chains. The MSDs of the terminal O_H3_ and C_V2_ atoms were calculated from the MD-generated trajectories. Example logarithmic graphs are reported in Figure 14. Only straight segments of the MSD lines in the time range of ca. 3 to 100 ns were analyzed. Curvatures for the times beyond ca. 100 ns are caused by accumulated statistical errors in the prolonged simulations. The exponents *α* in the power law for the MSD:

(1) $$\langle r^{2}\left(t\right)\rangle =K_{\alpha }t^{\alpha }$$

were calculated along with the generalized diffusion coefficients *K_α_* by the least squares method. The values reported in Table 5 for O_H3_ suggest that the polymer built has negligible if any effect on the exponent *α*. For this reason, we calculated the MSDs of C_V2_ for B13 and R13 only. The *α* values were 0.27 for the two dry polymers, and 0.29, 0.31, and 0.38 for those with water content of 10%, 20%, and 40%, respectively. The exponents slightly differed from one another for the systems with 60% of water. They were 0.42 for B13 and 0.43 for R13.

All reported *α* values are smaller than 1.00, evidencing the subdiffusion rather than Brownian motions for which *α* = 1.00. This semi-quantitatively agrees with the theory of polymer dynamics [60]. For the time range of intermediate lengths, such as the 100 ps in these simulations, the exponent *α* in Equation (1) is 0.50 for ideal chains and 0.54 for self-avoiding ones in the Rouse model [61]. Thus, the values of *α* for 60%-hydrated polymers approach that for the ideal chains. In Rouse’s approach, a polymer chain resembles beads connected by springs. Beads oscillate due to elastic forces and their motions are handicapped by friction against the surroundings. Water, however, acts as the lubricant and facilitates the movements of HEMA and VP side chains. Moreover, the free volumes are bigger in swelled hydrated polymers. Small water molecules do not clog up voids in the polymer material but rather move easily while pushed by the side chains.

The values of *α* for O_H3_ of HEMA chains in PHEMA, P(VP-*co*-HEMA), and P(MPC-*co*-HEMA) [26] are virtually the same and depend only on the water content in the simulated material. This evidences HEMA side chains insensitive to the molecular structure of the neighboring ones: short linear HEMA, long linear zwitterionic MPC, and short cyclic VP.

### 2.7. Dynamics of Water Molecules

**Figure 15:**
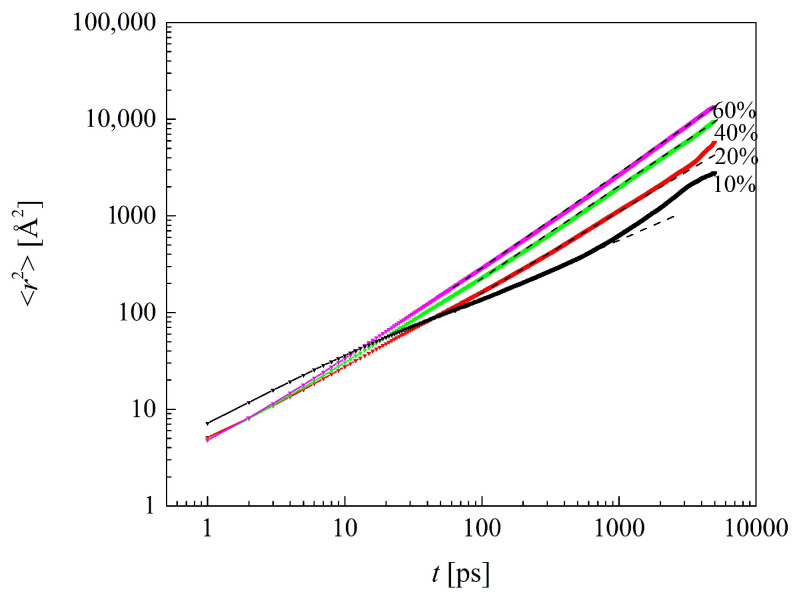
Mean square displacements of water molecules in hydrated H50 polymer as a function of time. Solid lines: simulation results; dotted lines: fitted Equation (1); line labels: mass percent of water in polymer material.

The dynamics of water molecules was analyzed in the same manner as that of polymer chains, except that the simulation time up to 1000 ps was considered. Examples of the MSD vs. time functions are plotted in Figure 15. The exponents *α* in the power law for the MSD, obtained by fitting the logarithmic form of Equation (1) to the simulated MSDs, are reported in Table 6.

The *α* < 1 evidences subdiffusion of water in low-hydrated hydrogels. Water molecules cram into the voids between the polymer side chains and hydrogen bond to the acceptor sites, hydroxyl O_H3_, and carbonyl O_H1_ and O_V_ in particular. In systems containing 40% and particularly 60% of water by mass, the fraction of “free” water molecules is sufficiently high for normal diffusion to occur, *α* ≈ 1. Differences between PHEMA and the four copolymers are rather small in this respect. VP mers in the polymer chain hamper slightly the molecular motions of water, resulting in the lowest *α* values for B13 and R13. This correlates with the glass transition temperature in the systems, which for B13 is the highest of these for the five dry polymers. However, the influence of the mer arrangement (block or random) on water diffusion is less pronounced than on the glass transition temperature. The values of *α* for water in P(VP-*co*-HEMA) are generally lower than those for P(MPC-*co*-HEMA) systems with the same water content. *α* for the latter reached 0.99 for copolymers of 37 HEMA and 13 MPC mers [26]. Thus, the VP mers in polymer chains impair the movements of water molecules stronger than HEMA mers and much stronger than long, flexible MPC mers.

Similarly to *α*, the diffusion coefficients of water in the polymers of the same water content are lower in P(VP-*co*-HEMA) and PHEMA than in P(MPC-*co*-HEMA) [26]. The diffusion coefficients D calculated from the MSDs are reported in Table 7. Again, the VP side chains lower the diffusion coefficient of water, contrary to the MPC ones. The copolymer configuration (block or random) is less, if at all, important. Be aware that the reported diffusion coefficients are just semi-quantitative values due to the rather short simulation time. Thus, they could be used with those reported earlier [26] in the trend analysis only, because of the same simulation procedure in the two studies. The rather obvious observation that polymer hydration facilitated water diffusion was also reported for other polymers [56,62].

**Figure 16:**
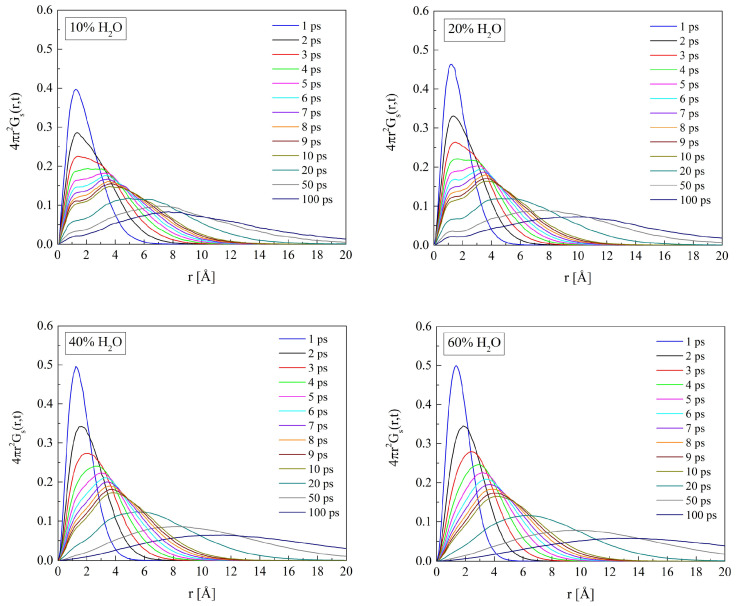
The self-terms of van Hove’s self-correlation functions $G_{s}\left(\vec{r},t\right)$ calculated for water molecules in H50 polymer with various water content.

**Figure 17:**
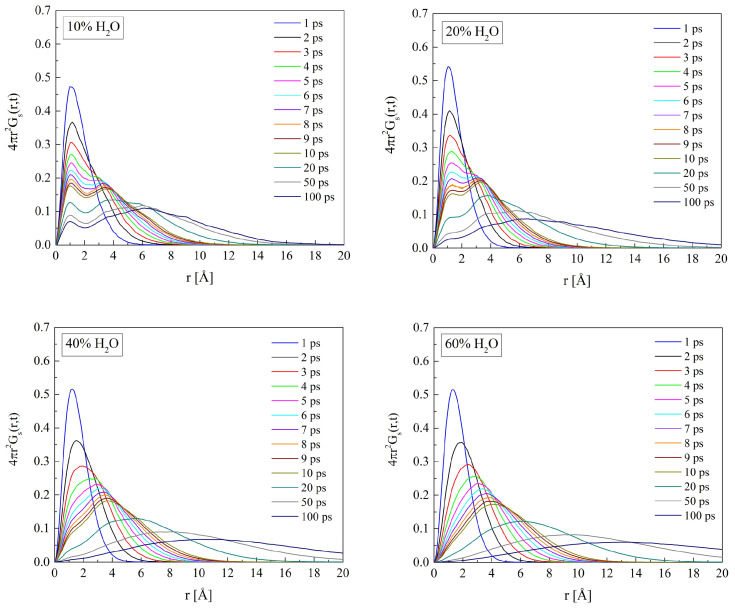
The self-terms of van Hove’s self-correlation function $G_{s}\left(\vec{r},t\right)$ calculated for water molecules in B13 copolymer with various water content.

The self-term of van Hove space-time correlation function $G_{s}\left(\vec{r},t\right)$ reflects water molecule movements in the polymer matrix. The $G_{s}\left(\vec{r},t\right)$ for O_W_ in hydrated H50 and B13, calculated for the time interval from 1 to 100 ps, are shown in Figure 16 and Figure 17. For short simulation time, the distribution of displacements is unimodal. Single peaks evidence molecular vibrations within “cages” in the material. The sharper the peak, the more even the amplitude of the oscillations about the equilibrium point. In a longer time, the molecule can travel farther away from its initial position because dynamic changes in its surroundings may decrease the energy barrier between the adjoining sites of the minimum potential energy. If the jumps from one equilibrium point to another are scarce in comparison with the oscillation frequency of the molecule, another peak or peaks appear on the $G_{s}\left(\vec{r},t\right)$ function. Therefore, the multimodal distribution of intermolecular distances suggests the molecules “hopping” from one cage to another. The $G_{s}\left(\vec{r},t\right)$ functions calculated for water molecules in PHEMA and P(VP-*co*-HEMA) show up to two peaks. Two distinct peaks occur for the systems with 10 and 20% of water by mass. In highly hydrated systems, the “hopping” vanishes and the diffusion of free water predominates. The self-correlation function for such systems is rather flat with a single maximum at distances significantly exceeding the hydrogen bond length. In this respect, the PHEMA and P(VP-*co*-HEMA) hydrates do not differ from those of P(MPC-*co*-HEMA) reported earlier [26].

## 3. Computer Simulations

In the previous work, we reported definitions and simulation details [26]. For readers’ convenience, we repeat here pieces of information necessary for the interpretation of the results obtained in the present study.

### 3.1. Simulation Procedure

In the molecular dynamics (MD) simulations, we applied the Forcite and Amorphous Cell modules of the Materials Studio package [63]. The geometry of the simulated polymer chains was optimized with the Smart method implemented in the Forcite module. A 5 ns long isothermal–isochoric (NVT) MD simulation with a time step of 1 fs that evidenced the system equilibrated thermodynamically after 3 ns. The maximum number of geometry optimization cycles and the energy convergence criterion were equal to 1000 and 0.001 kcal/mol, respectively. The Dreiding force field was used to perform MD simulations [26,64]. The MD simulations were controlled by a Nosé–Hoover thermostat [65] set to a temperature of 307 K, i.e., that of the eyeball surface [66,67,68]. Polymer configurations obtained in this way were the starting ones in MD simulations of hydrates.

In the following step, the polymers were hydrated up to the 60% swelling ratio using the Amorphous Cell module. To avoid lengthy calculations, only one polymer chain occupied each unit cell. The unit cell size was adjusted to the final density of the system equal to 1.1 g/cm^3^. Calculations were similar to those applied in the first step, except that the electrostatic and van der Waals interactions were calculated by the 3D Ewald summation.

### 3.2. System Volume

The polymer volumes for the glass transition temperature assessments were calculated in the isothermal–isobaric (NPT) MD simulations. First, the temperature was set to 650 K and then dropped by 25 K steps until it reached 200 K. At each temperature, the systems were equilibrated at 1 atm for 200 ps. The simulations were controlled by the Nosé–Hoover thermostat [65] and Andersen barostat [69].

### 3.3. Functions and Quantities

Hydrogen bonds (D-H···A) fulfilled the following criteria: donor–acceptor atom distance (D···A) of 3.5 Å or shorter, acceptor–hydrogen donor atom distance (H···A) less than 2.5 Å and donor hydrogen–acceptor atom (D-H···A) deviation from linearity up to 30° [70,71].

The free volumes were estimated using the Connolly surface method by probing the free spaces with a sphere of a given radius [72]. The van der Waals radii of the atoms forming polymers were r_C_ = 1.70 Å, r_H_ = 1.20 Å, r_O_ = 1.52 Å, and r_N_ = 1.55 Å. The fractional free volume (FFV) is the ratio of free volume to the total volume of the system [73].

The diffusion coefficient, D, was obtained from the mean square displacement (MSD), $\langle r^{2}\left(t\right)\rangle$, using Einstein’s relation:

(2) $$D=\frac{1}{6N}\underset{t\to \infty }{\lim}\frac{d}{dt}\langle r^{2}\left(t\right)\rangle ,$$

where *N* is the number of diffusive atoms in the system, and

(3) $$\langle r^{2}\left(t\right)\rangle =\frac{1}{N}\sum_{i=1}^{N}{\left|\vec{r_{i}}\left(t\right)-\vec{r_{i}}\left(0\right)\right|}^{2},$$

where $\vec{r_{i}}\left(0\right)$ and $\vec{r_{i}}\left(t\right)$ are the initial and final positions of the *i*-th particle over the time interval *t*, and *N* is the number of equivalent particles.

The van Hove space-time correlation function, $G\left(\vec{r},t\right)$, defined as the correlation between the position of the particle of ${\vec{r}}^{\prime }+\vec{r}$ at the time $t^{\prime }+t$ and its position of ${\vec{r}}^{\prime }$ at the time $t^{\prime }$, decomposes into two parts: the self-term $G_{s}\left(\vec{r},t\right)$ and distinct term $G_{d}\left(\vec{r},\;t\right)$. In this study, we analyzed the self-terms for water molecules in the hydrated polymers. $G_{s}\left(\vec{r},t\right)$ characterizes the distribution of displacements at time *t*. The second moment of this distribution and MSD relate to each other in the following manner:

(4) $$\langle {\left[\vec{r}\left(t\right)-\vec{r}\left(0\right)\right]}^{2}\rangle =\int r^{2}G_{s}\left(\vec{r},\;t\right)d\vec{r}.$$

## 4. Conclusions

Both PHEMA and P(VP-*co*-HEMA), dry and hydrated, were amorphous materials in these simulations. HEMA and VP side chains are relatively rigid. The geometry and dynamics of the HEMA side chain are insensitive to the neighboring chains: short linear HEMA and short cyclic VP. The VP side chains are stiffer than the HEMA ones. The more VP mers in the P(VP-*co*-HEMA) chain, the higher the glass transition temperature of the dry polymers. The effect is bigger for block copolymers. Copolymerization does not affect the distances between the selected atom pairs, regardless of sequence and number of mers in the polymer chain. Side chains show subdiffusion rather than normal diffusion. Water enhances their mobility.

In hydrated polymers, water penetrates the voids merged into diffusion channels. As long as water concentration is below 40% by mass, much of it is hydrogen-bonded to the polymer. Water molecules form hydrogen bonds with the hydroxyl and carbonyl groups of HEMA. Hydrogen bonds with the ether O_H2_ are scarce. In VP side chains, the main hydrogen bonding site is the carbonyl O_V_. Water does not form hydrogen bonds with N_V_ atoms because the main and side polymer chains hinder access to the lone-pair electrons. As a consequence of this hydrogen bonding, water molecules show subdiffusion because of the restricted freedom of motion. The process involves the “hopping” of water molecules from one local minimum of the potential energy of molecular interactions to another. Between the “hops”, the molecule oscillates at the minimum.

The VB side chains lower both the exponents α in the power law for the MSD and the diffusion coefficient of water. Thus, the effects of VP side chains in copolymers with HEMA on the mobility of water molecules are opposite to one another. For higher concentrations of water (40 and 60%), the normal diffusion becomes pronounced because of the increased share of water molecules non-hydrogen-bonded to the polymer.

In summary, PHEMA and P(VP-*co*-HEMA) hydrogels demonstrate properties suitable for use in ophthalmic devices, particularly in contact lenses where sustained hydration and water transport are necessary. PHEMA may offer advantages in water retention and molecule diffusion due to stronger polymer–water interactions, whereas the incorporation of VP in the polymeric chain contributes to hydration but may slightly impede water diffusion. Thus, while both hydrogels hold promise, further optimization, particularly of P(VP-*co*-HEMA) configurations, may enhance their performance for contact lens applications where moisture, comfort, and permeability are essential.

## Figures and Tables

**Table 1:** Calculated glass transition temperatures *T*_g_ of non-hydrated polymers.

Polymer	*T*_g_ [K]
H50	425
B37	460
B13	468
R37	450
R13	457

**Table 2:** The cell sizes for the PHEMA and P(VP-*co*-HEMA) after 5 ns long MD simulations.

Polymer	Water Content by Mass (%)	Number of Water Molecules in the Cell	Unit Cell Edge (Å)
H50	0	0	21.434
	10	40	22.196
	20	90	23.080
	40	241	24.592
	60	543	29.088
B37, R37	0	0	21.160
	10	39	21.922
	20	87	22.792
	40	232	25.084
	60	522	28.712
B13, R13	0	0	20.635
	10	36	21.374
	20	81	22.232
	40	215	24.459
	60	484	27.999

**Table 3:** The fractional free volumes (FFVs) in PHEMA and P(VP-*co*-HEMA) of different built and degrees of hydration for the probe of infinitesimal radius.

Water Content (%)	FFV (%) of the Polymer				
	H50	B37	B13	R37	R13
0	38.3	38.2	37.9	38.2	37.7
10	37.5	37.6	36.9	37.5	37.0
20	36.5	36.9	36.6	37.0	36.3
40	35.9	35.8	35.6	35.7	35.5
60	34.9	34.8	34.5	35.0	34.7

**Table 4:** The fractional free volumes (FFVs) in PHEMA and P(VP-*co*-HEMA) available for water molecules, calculated for a spherical probe of 1.4 Å radius.

Water Content (%)	FFV (%) of the Polymer				
	H50	B37	B13	R37	R13
0	10.4	11.3	12.5	10.0	10.8
10	26.5	22.1	19.6	21.6	22.6
20	34.5	31.8	31.5	32.2	31.3
40	51.7	50.9	50.2	50.9	52.5
60	68.4	67.8	68.9	68.3	67.9

**Table 5:** The exponents *α* in the power law for the MSD (Equation (1)) of the O_H3_ atom of polymers with various water content.

Water Content (%)	*α* (O_H3_)				
	H50	B37	R37	B13	R13
0	0.28	0.26	0.25	0.27	0.27
10	0.30	0.31	0.31	0.30	0.30
20	0.34	0.33	0.34	0.34	0.34
40	0.34	0.34	0.33	0.34	0.33
60	0.44	0.43	0.44	0.44	0.44

**Table 6:** The exponents *α* in Equation (1) for MSD of water molecules in hydrated P(VP-*co*-HEMA) copolymers.

Water Content (%)	*α* (H_2_O)				
	H50	B37	R37	B13	R13
10	0.69	0.72	0.72	0.71	0.70
20	0.89	0.85	0.85	0.82	0.80
40	0.93	0.93	0.91	0.91	0.90
60	0.97	0.96	0.96	0.96	0.95

**Table 7:** Diffusion coefficients of water molecules in hydrated PHEMA and P(VP-*co*-HEMA).

Water Content (%)	*D* ∙ 10^−5^ [cm^2^ s^−1^]				
	H50	B37	R37	B13	R13
10	1.15	1.20	1.20	1.18	1.16
20	1.48	1.42	1.42	1.35	1.33
40	1.55	1.55	1.52	1.52	1.50
60	1.61	1.60	1.60	1.58	1.58

## Data Availability

Computationally obtained data available from K.F.-S. on personal request.
